# RbQE: An Efficient Method for Content-Based Medical Image Retrieval Based on Query Expansion

**DOI:** 10.1007/s10278-022-00769-7

**Published:** 2023-01-26

**Authors:** Metwally Rashad, Ibrahem Afifi, Mohammed Abdelfatah

**Affiliations:** 1 Department of Computer Science, Faculty of Computers & Artificial Intelligence, Benha University, Benha, Egypt; 2 Faculty of Artificial Intelligence, Delta University for Science and Technology, Gamasa, Egypt; 3 Department of Information System, Faculty of Computers & Artificial Intelligence, Benha University, Benha, Egypt

**Keywords:** Medical image retrieval, Pre-trained learning models, Query expanded, Mean value, Deep features

## Abstract

Systems for retrieving and managing content-based medical images are becoming more important, especially as medical imaging technology advances and the medical image database grows. In addition, these systems can also use medical images to better grasp and gain a deeper understanding of the causes and treatments of different diseases, not just for diagnostic purposes. For achieving all these purposes, there is a critical need for an efficient and accurate content-based medical image retrieval (CBMIR) method. This paper proposes an efficient method (RbQE) for the retrieval of computed tomography (CT) and magnetic resonance (MR) images. RbQE is based on expanding the features of querying and exploiting the pre-trained learning models AlexNet and VGG-19 to extract compact, deep, and high-level features from medical images. There are two searching procedures in RbQE: a rapid search and a final search. In the rapid search, the original query is expanded by retrieving the top-ranked images from each class and is used to reformulate the query by calculating the mean values for deep features of the top-ranked images, resulting in a new query for each class. In the final search, the new query that is most similar to the original query will be used for retrieval from the database. The performance of the proposed method has been compared to state-of-the-art methods on four publicly available standard databases, namely, TCIA-CT, EXACT09-CT, NEMA-CT, and OASIS-MRI. Experimental results show that the proposed method exceeds the compared methods by 0.84%, 4.86%, 1.24%, and 14.34% in average retrieval precision (ARP) for the TCIA-CT, EXACT09-CT, NEMA-CT, and OASIS-MRI databases, respectively.

## Introduction

**Fig. 1:**
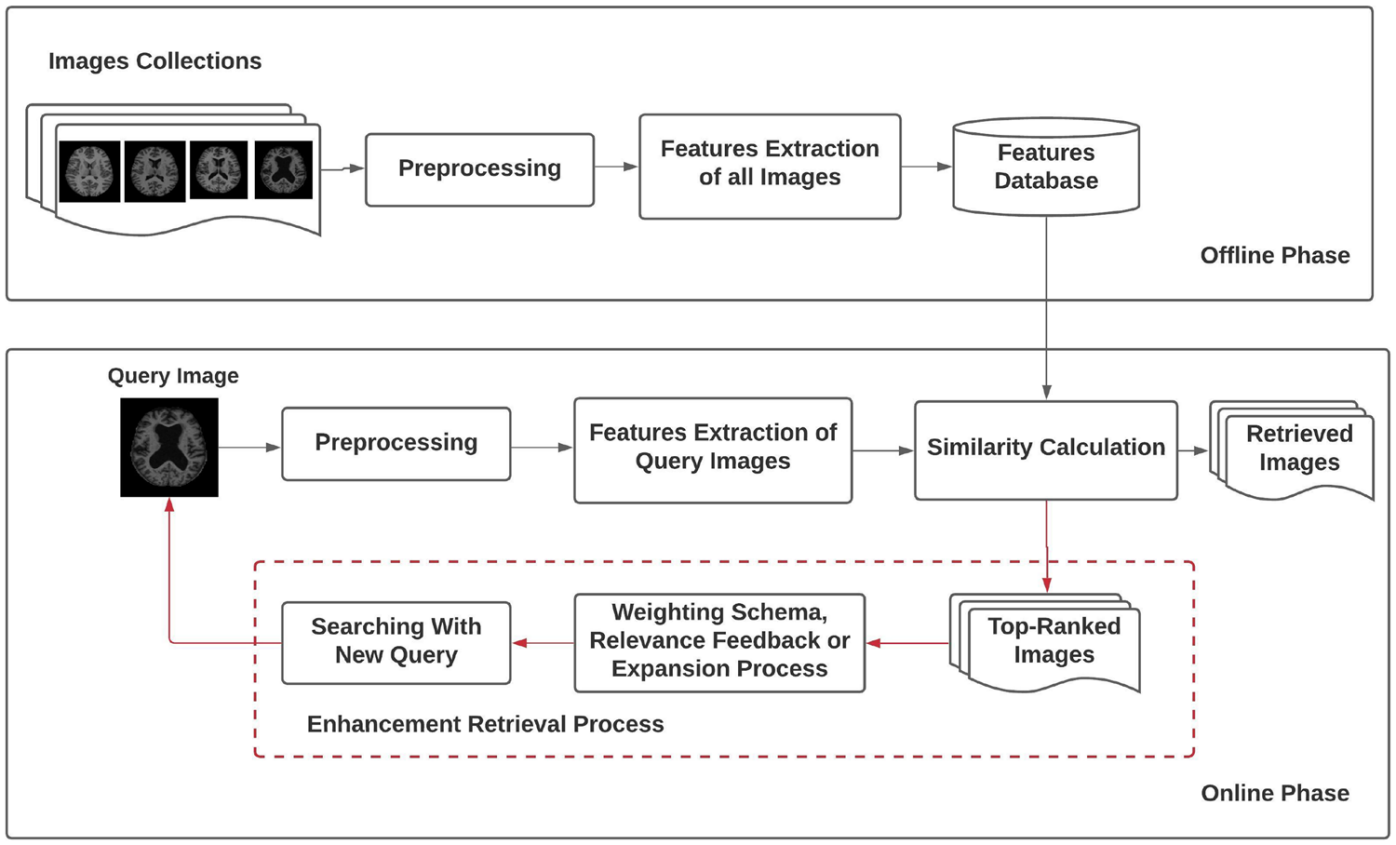
The CBMIR Main Architecture

One of the most active medical image processing research domains, according to recent studies, is content-based medical image retrieval (CBMIR). This is because the usage of several medical techniques, including ultrasound (US), MR, X-ray, and CT, is expanding and accelerating. The similarity of the images is considered the most important thing that CBMIR’s systems are focused on. The user submits a query, and the system retrieves images with the same criterion of similarity in descending order. The two fundamental steps of every CBMIR technique are feature extraction (offline phase) and similarity measurement computations (online phase) [1–3]. The CBMIR system’s main architecture is shown in Fig. 1. The CBMIR system has many upgrades that were created to improve its effectiveness and retrieval performance, which can be at the stage of pre-processing or extraction [4, 5]. The extensive medical image retrieval literature shows that texture-based features are well-accepted and popular among researchers worldwide [6–10]. However, medical imaging becomes more sophisticated over time as it attempts to gather as much information about the patient’s anatomy as possible. As a result, developing a powerful CBMIR system based solely on texture is insufficient. It is, therefore, necessary for the hour to build a system for the multi-dimensional retrieval of medical images that will combine multi-dimensional information, for example, texture, edge, and shape. It is a fundamental component of any CBMIR system that compares an image to a database image to determine how similar they are and to find matching pairings for the image [11, 12]. Traditional methods rely on low-level extraction by assessing their colors, textures, forms, and spatial structure from medical imagery. All features are low-level and often do not accurately reflect semantic notions in the images. Using these features for retrieval usually yields unsatisfactory results. Therefore, pre-trained deep convolutional neural network (DCNN) model features have lately achieved superior performance and flexibility than classical descriptors in common image retrieval applications due to the quick advancement of deep learning (e.g., image retrieval or object recognition). Rich image semantic information is provided by this feature, which is crucial for improving the precision of image retrieval.

Having considered all of this in mind, in this paper, the RbQE approach has been used to demonstrate an effective way to retrieve CT and MR images. The RbQE expands the query image by reformulating it based on calculating the mean value of the top-ranking images from each class, and this expansion method is considered fully automated. The RbQE method benefits from the pre-trained DCNN (AlexNet and VGG-19) as extractors of features that are compact, high-level, and robust toward image noise to best represent the medical images and achieve high accuracy. The main contributions of our paper are summarized as follows:

1. Proposed an efficient RbQE medical image retrieval method that expands the query in a new automated way.
2. Use the pre-trained deep convolutional neural networks (AlexNet and VGG-19) as feature extractors that describe and represent medical images to obtain complex and high-level features, which have the best ability to withstand external interferences, such as changes in lighting, noise, rotation, and blurred images.
3. Extensive tests were carried out to compare the performance of the proposed method (RbQE with DCNN) with the existing and modern methods, and it demonstrated that the proposed method exceeds all these methods in retrieving medical images.

The remainder of the paper is arranged as follows. The literature review is shown in the “Literature Review’’ section. The proposed method is described in the “Proposed Method’’ section. The “Experimental Framework’’ section describes the experimental framework used to evaluate the performance of the retrieval to the proposed method and comparative methods. The experimental results of the proposed method and all comparative methods are presented in four standard medical databases in the “Experimental Results’’ section. Finally, conclusions are presented in the “Conclusion’’ section.

## Literature Review

Content-based medical image retrieval (CBMIR) technology has a very important role in medical image analysis, where the existing CBMIR technology is used to index and retrieve medical images by using traditional visual indicators to represent all medical images in the image database. Standard descriptors of visual content include texture, edge, color histogram, shape, and a large number of variants. In the past, CBMIR feature extraction was a critical aspect of the accurate retrieval of medical images. CBMIR strives to remove redundant information by reducing the dimensionality of image data [13, 14].

Medical images are available in different formats, such as CT and MRI images. As a result, the authors of [15] proposed a method for detecting CT and MRI co-occurrences based on local feature descriptors. The authors in [16] present a robust and fast MRI retrieval system for brain images. A powerful textural descriptor, known as the local binary pattern (LBP), was proposed in [17]. Based on the LBP, the feature vector is created by each pixel’s intensity. The authors in [18] use LBP, joint LBP, and histograms of image intensity to produce a pulmonary emphysema quantitative analysis of CT. In [19], the feature extractor technology was principally used to evaluate CT images of the chest on the basis of structure and local brightness. The authors in [20] presented a local ternary co-occurrence pattern (LTCoP). In [21], they proposed a BMI approach known as the local mesh pattern (LMeP), and the approach provided in [22] established an LMePVEP algorithm.

For high-level feature descriptors, in [23, 24], the authors present some studies on the ability to obtain efficient images using convolutional neural networks (CNNs), which have been used in machine learning applications. The authors suggested in [25] the AlexNet descriptor medical image retrieval system for local bit plane decoding (LBpDAD), which combines the benefits of local bit plane decoding with the features resulting from a neural network like AlexNet. The authors in [26] introduced the histogram of compressed scattering coefficients (HCSCs) method, where they created a new feature based on employing the transformation in the scatter and a specific version of deep networks to determine the textural features of CT images. Furthermore, [27] proposed an integrated scattering feature based on two separate forms of compressed scattering data: data concentration and canonical correlation analysis (CCA). The authors presented an image reconstruction network (IR-Net) in [28], where the input image would be encoded into a set of features before being rebuilt from the encoded features.

When it comes to the expand query approach, the expansion gains from the label data of the top-ranked images that are obtained and saved in a feedback session. In the literature, there have been numerous successful attempts at various expansion techniques depending on local, global, and CNN features, including certain functionalities that include expanding queries and other methods and a recent query expansion review in data retrieval [29]. The authors in [30] have broken down the expansion model into two components: offline and online retrieval. In the offline procedure, the Laplacian score method is generalized for computation, while the query is classified according to the feature score of the relevant items in the online retrieval component of the database. Finally, the original query was replaced by a slew of first-page results. Their tests with sets of images and single-voice objects were far superior to those of their opponents.

The authors in [31] introduced the query expansion approach, where they used the pre-trained CNN model by using the convolutional layer’s learning filters as visual word detectors. Combined with geometric testing, query expansion techniques are particularly effective in the context of using top-relevant images to expand query-relevant features into eventual successful and valid matches, as shown in [32].

The authors of [33] attempted to extend the automated query expansion by proposing three extensions, where the spatial verification was improved and repositioning was done by reflecting the previously evaluated results, and suggested an approach that expands the query by integrating matching features outside the original query limit, utilizing the spatial context. The authors in the latest study [34] have created a query expansion template based on the mathematical architecture by treating query extension as a discrimination-related learning issue, in which a grouping model is supervised and learned, and then (LAttQE) offered the addition model to communicate data through automatic attention between the top-ranking item and the query. The top-ranked techniques are increasingly used; in the case of building a framework for multimodal query expansion through user interaction methods [35], several approaches are used to achieve this goal.

The authors in [36] achieved a significant level of accuracy in the retrieval of MRI and histopathological images by introducing an expansion approach for features extracted by pre-trained Residual Networks (ResNets).

## Proposed Method

**Fig. 2:**
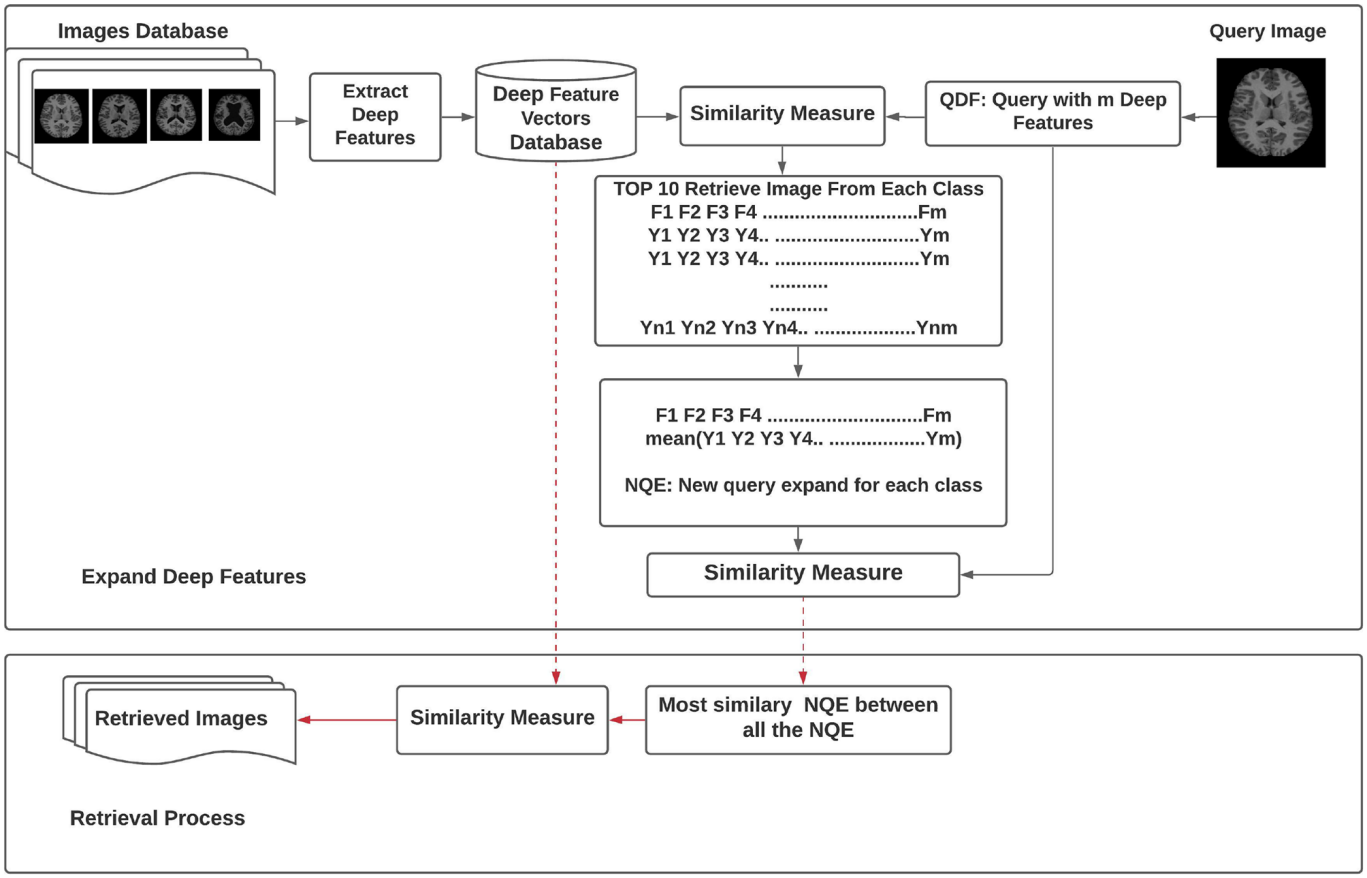
Illustration of the RbQE method

The proposed method has two important parts, which are shown in Fig. 2. The first part is an excellent feature extractor, and the second is an efficient matching and retrieval method for medical images. So, two deep feature extractors and the RbQE (retrieval based on query expansion) method have been used in the proposed method. Based on pre-trained models, the deep feature extractor can extract compact and high-level features to represent all images in the medical database. There are two aims of using the deep neural network instead of raw pixels in the analysis of medical images: the first is to extract invariant features, which are more robust against different interferences like noise and changes in the light that appears during the generation of the medical images. Second, there is no need for the deep feature extractor to be retrained, if trained offline using a huge image database, even in the case of analyzing various types and formats of medical images. Consequently, the used deep model is likely to dramatically increase computational efficiency and lower calculation costs in comparison to other retrieval systems that also use deep models.

In addition, the RbQE method is used to improve the matching and retrieval in the CBMIR by expanding the deep features of the original query and the construction of a new query. The RbQE method relies on two search processes: a quick search and a final search. First, a rapid search of the database will retrieve the top-ranked images for the original query from each database class, and for each class, a new query expansion (NQE) will be formed. Secondly, in the final search, the image that is most similar (NQE) to the query images is taken and used as the final new query expansion (FNQE), which is one of the main benefits of our suggested method. The next subsections provide more information on these feature extractors and query expansion methods.

### Deep Feature Extraction

We use more robust and efficient deep features to extract more discriminative and high-level features for medical images, thereby minimizing the interference problem. Deep learning has gained enormous popularity recently, with promising applications in a variety of areas [37]. The basic idea behind deep learning has not changed, despite the fact that numerous architectures have been proposed and put into practice: deep learning is a feature representation learning approach that concentrates on huge amounts of unprocessed image data and can use different levels of representation. This concept is stable in spite of several models of deep learning that have been suggested and implemented. Many levels of abstraction enable learning data representations by computational models with many layers of processing L ($L>1$), where after the input layer, each layer transforms the representation of the preceding layer into a more abstract representation, then you can obtain complex structures indirectly from large format imagery and ideally use them to create the original image or the image of the query after studying most of the distinctive variations layer by layer.

**Fig. 3:**
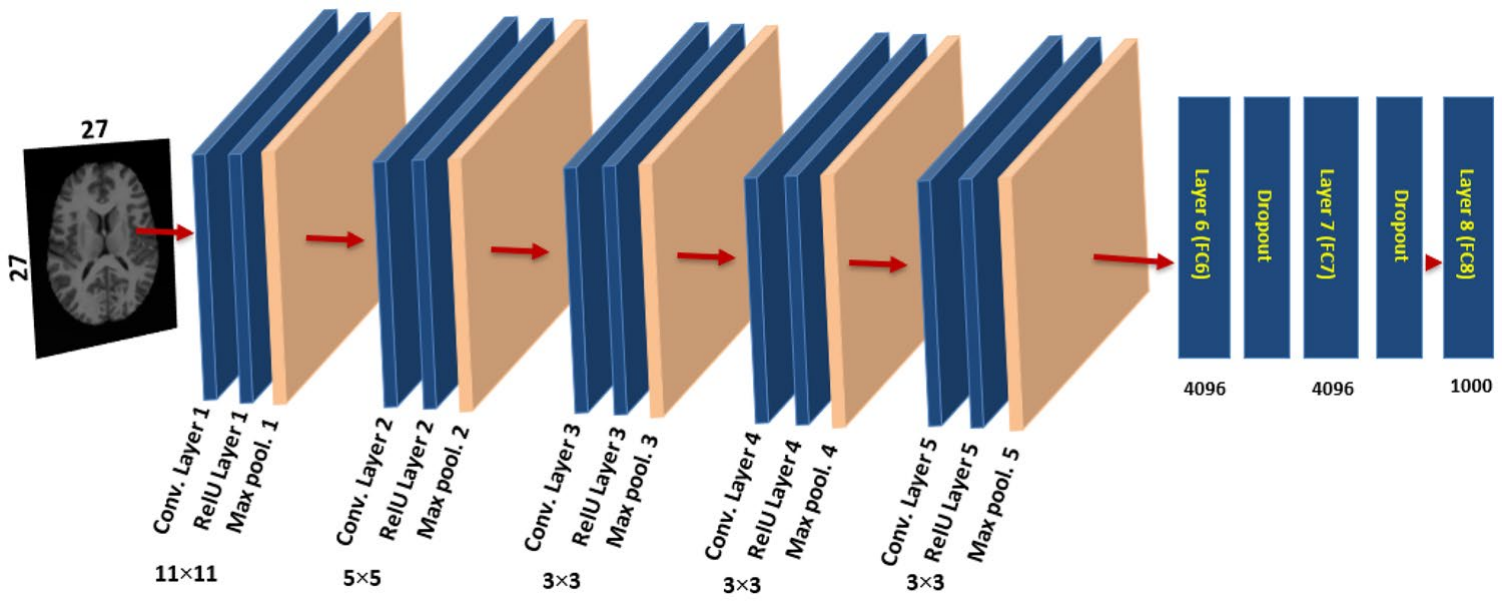
The pre-trained CNN (AlexNet) on ImageNet database

**Fig. 4:**
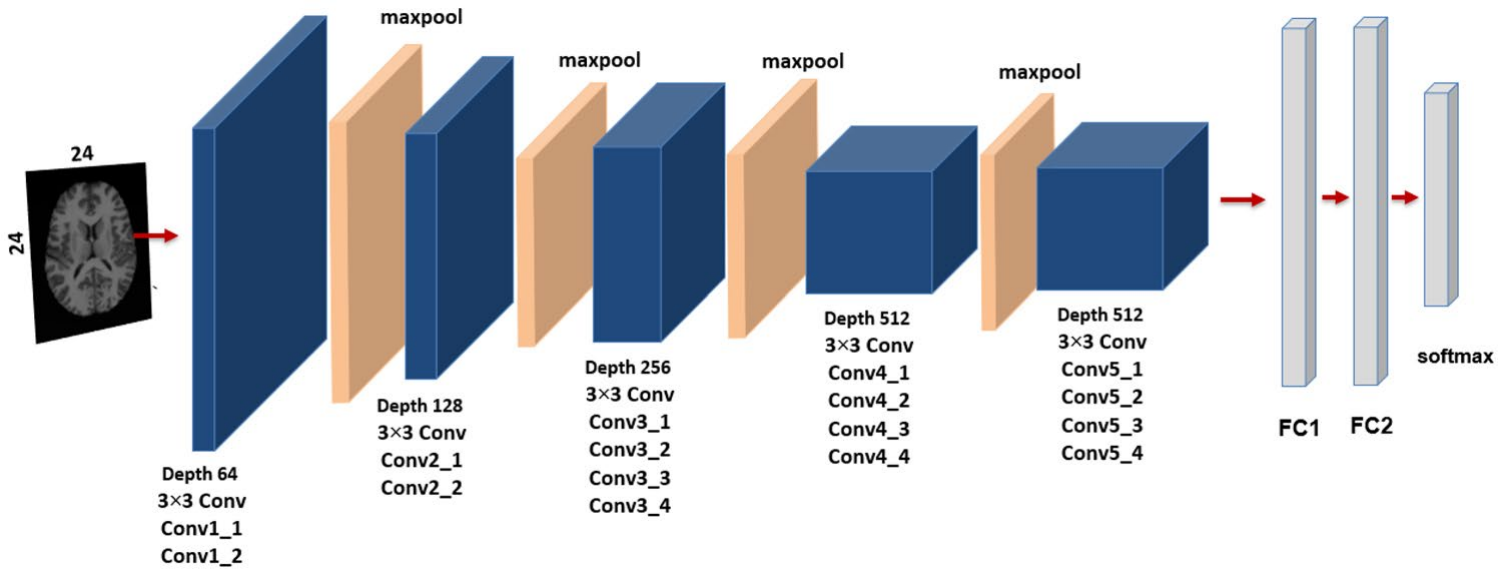
The pre-trained DCNN (VGG-19) on ImageNet database

In this paper, for medical image retrieval, two types of supervised CNN learning models are used as deep neural networks. CNN is a form of neural network that has been proposed to deal with images and obtain local features located in images. To work with high-resolution images, CNN has three properties: First, each convolution kernel has a small function in depth that is a feature that, despite its small size, can distinguish between different images. Second, since each convolutional feature map uses the same convolution kernel, the same deep features may be filtered and obtained from different locations in the input image. Finally, by subsampling from the convolutional layer to the pooling layer, the image’s dimensionality is reduced and computing efficiency is increased. Figure 3 shows a previously trained DCNN model (AlexNet) and Fig. 4 shows other previously trained DCNN models (VGG-19), which have been trained offline in the ImageNet database [38] and contain millions of labelled images.

Significantly deeper neural networks cannot be used for medical image processing. Because the small differences between identical biomedical images with high-level features are difficult to differentiate, the small disparity will disappear with greater abstraction. However, a small difference is particularly essential in biological images and may be applied precisely to discriminate biomedical images of several types, such as images from our research in the OASIS-MRI database used in our research. As shown in Fig. 3, the AlexNet, which is inspired by biological processes in which the object is recognized from the low-level to the semantic level step by step, is typically composed of four key components: Firstly, the convolutional layers, which are connected to a limited, mostly human visual system location by a convolutional kernel and considered the greatest highlight of AlexNet. Secondly, the activation functions are frequently followed by the convolutional layers, where the ReLU (rectified linear unit) activation function is used to extract from the input signals the more complicated features. Thirdly, the dimensionality of the feature map is lessened by the pooling layers, while the convolutional layer sensitivity is decreased. Finally, at the conclusion of the AlexNet structure, the fully connected layer is combined to generate a feature vector, which provides the prediction result. By applying the backpropagation approach, the loss function between the prediction outcomes and ground truth is minimized using the AlexNet training procedure until the error loss is considerably reduced or a certain number of iterations have been completed. We have used the learned AlexNet as an extractor of biomedical frameworks, utilizing the fully connected layer-6. We use completely connected layer-6 features, since various studies have shown that layer-6 features are more efficient than layer-7 features in biomedical image processing [39–43].

Convolutional networks with extremely deep layers (up to 19 weight layers) (VGG-19) were employed as a feature extractor in our paper, where there are 16 convolutional layers and 3 FC layers, as shown in Fig. 4, where the number of channels is quite small, starting at 64 in the first layer and growing by a factor of two after each max-pooling layer until it reaches 512. In this network (VGG-19), the image was transmitted through a stack of a convolutional layer that is a composite of filters with an extremely narrow receptive field $3 \times 3$ to gripe the notion of up/down, left/right, and center. The convolution stride was set to one pixel, and the spatial padding of convolution is 1 pixel $3 \times 3$ convolution layers. There are five max-pooling layers, each of which was conducted across a $2 \times 2$ pixel window with stride 2. Three Fully Connected (FC) layers follow a stack of convolutional layers: the first two (FC1, FC2) have 4096 channels (features) apiece, while the third (FC3) has 1000 channels (features), and the soft-max layer is found in the final. Also, here we have utilized the fully connected layer-1 (FC1) of the VGG-19 as a feature vector extractor. There are also various studies that show that FC1 features are more efficient than layer-2 (FC2) features in biomedical image processing, except in the TCIA-CT database, where FC2 features have achieved higher accuracy than FC1 features.

### Query Expansion Method

**Fig. 5:**
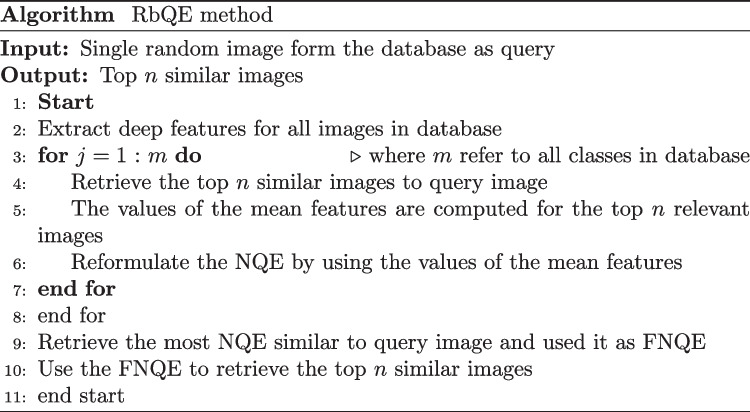
RbQE method algorithm

As shown in Fig. 2, which describes the complete idea of the expansion of deep features for the original query and the reformulation of a new query for the final search process, the RbQE technique employs the mean values of the deep feature values for images of the top-ranked after a rapid search using a “Query with 4096 Deep Features” (QDF) of the original query to all deep features of photos in the database. From each class in the database, the top ten similar images to the original query are retrieved, and the mean value of deep features for each of the top ten is calculated. This process produces a number of NQEs equal to the number of classes in the database. After that, the most similar NQE to the original query will be taken as the final NQE (FNQE), and then the FNQE is used for the final search procedure. Table 1 provides a simple numerical example of building NQE, where the feature vector dimension for each image is 4096 for both AlexNet and VGG-19. Figure 5 illustrates the proposed algorithm for the RbQE method.

**Table 1:** NQE based on mean values

	F1	F2	F3	F4	F5	$\cdots \cdots$	F4096
Img1	0.99	-15.05	-5.02	-41.11	-23.11	$\cdots \cdots$	6.76
Img2	2.21	–12.71	–3.55	–43.14	–23.29	$\cdots \cdots$	8.57
Img3	–1.29	–13.40	–7.85	–39.39	–28.45	$\cdots \cdots$	8.29
Img4	–1.48	–11.07	–3.12	–37.64	–23.31	$\cdots \cdots$	13.11
Img5	–6.69	–7.70	1.03	–40.86	–22.97	$\cdots \cdots$	6.07
NQE	–1.252	–11.986	–3.702	–40.428	–24.226	$\cdots \cdots$	8.56

## Experimental Framework

This section presents the computational methods used to compare the performance of the proposed method with other modern retrieval methods. The name and abbreviations of all methods used for comparison with the proposed method are presented in Table 2.

**Table 2:** Name and Abbreviations of all methods used in the comparison

S. No.	Abbreviation	Method Name	Reference	Year
1	LBP	Local Binary Pattern	[17]	1996
2	LTP	Local Ternary Pattern	[44]	2010
3	LDP	Local Derivative Pattern	[45]	2010
4	LTrP	Local Tetra Patterns	[46]	2012
5	AlexNet	Deep Convolutional Neural Networks	[47]	2012
6	LTCoP	Local Ternary Co-Occurrence Patterns	[20]	2013
7	LMeP	Local Mesh Patterns	[21]	2014
8	VGG-16	Visual Geometry Group	[48]	2014
9	LWP	Local Wavelet Pattern	[49]	2015
10	SS3D	Spherical Symmetric 3D Local Ternary Patterns	[50]	2015
11	ResNet	Residual Neural Network	[51]	2016
12	HCSCs	Histogram of Compressed Scattering Coefficients	[26]	2017
13	ST-CCA	Scattering Transform with Canonical Correlation Analysis	[27]	2018
14	MDMEP	Multi-dimensional multi-directional mask maximum edge pattern	[52]	2018
15	IR-Net	Image Reconstruction Network	[28]	2020

### Image Similarity Estimation

Similarity values are calculated with the Euclidean Distance (ED), which is used to calculate the similarity, for both rapid and final search. Let X = ($x_{1}$, $x_{2}$,..., $x_{n}$) and Y = ($y_{1}$, $y_{2}$,..., $y_{n}$), two feature vectors with n dimension, the similarity is computed as follows:

1 $$\begin{aligned} ED \left( X,Y\right) =\sqrt{\sum \limits _{i=1}^{n} \left( x_{i}-y_{i}\right) ^2} \end{aligned}$$

### Performance Estimation

In experiments, every image in the database is used as a query, and an image is only relevant if it belongs to the same category as the query. Average Precision Retrieval (ARP), Average Retrieval Rate (ARR) and $F_{score}$ are the three performance metrics used to evaluate each retrieval strategy.

2 $$\begin{aligned} precision:P(q)=\frac{\mathrm{Number\;of\;relevant\;images\;retrieved}}{\mathrm{Number\;of\;images\;retrieved}} \end{aligned}$$

3 $$\begin{aligned} recall:R(q)=\frac{\mathrm {Number\;of\;relevant\;images\;retrieved}}{\mathrm{Number\;of\;relevant\;images\;in\;the\;database}} \end{aligned}$$

4 $$\begin{aligned} ARP (\%)=\frac{100}{\mid DB \mid }\sum \limits _{i=1}^{\mid DB \mid } P(I_i) \end{aligned}$$

5 $$\begin{aligned} ARR (\%)=\frac{100}{\mid DB \mid }\sum \limits _{i=1}^{\mid DB \mid } R(I_i) \end{aligned}$$

6 $$\begin{aligned} F_{score} (\%)=\frac{2 \times \text {ARP}\times \text {ARR}}{\text {ARP}+ \text {ARR}} \end{aligned}$$

where $\mid DB \mid$ indicates the count of all database images.

### Image Model Databases

**Fig. 6:**
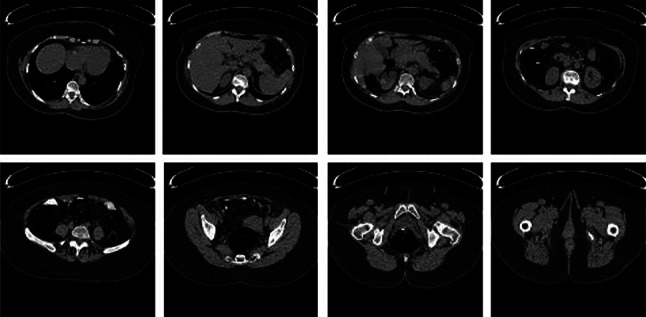
Sample images from each class of TCIA-CT database

**Fig. 7:**
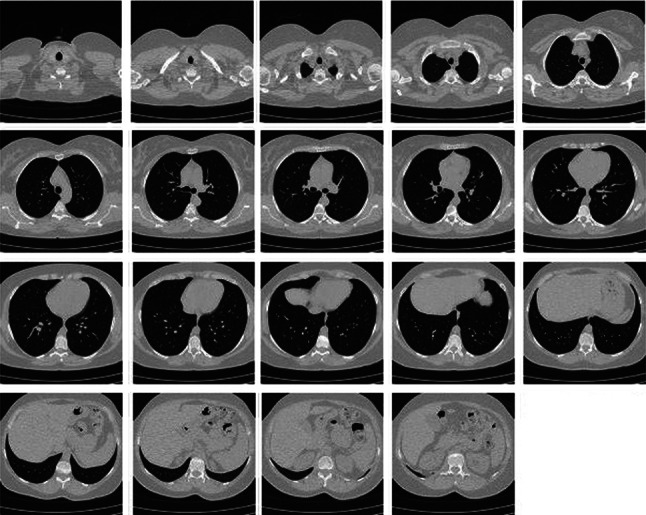
Sample images from each class of EXACT09-CT database

**Fig. 8:**
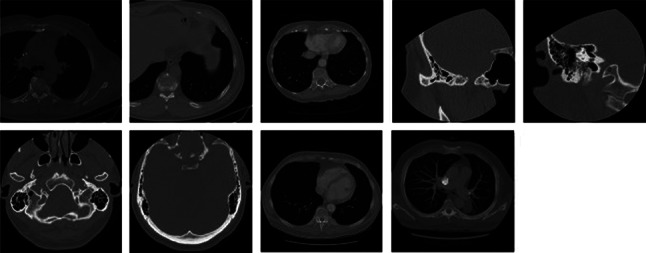
Sample images from each class of NEMA-CT database

**Fig. 9:**
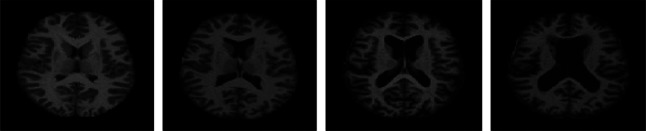
Sample images from each class of OASIS-MRI database

Experiments were carried out on four publicly available image databases with different formats in order to test the performance of the RbQE method, namely the TCIA-CT database [53], the EXACT09-CT database [54], the NEMA-CT database [55] for CT image retrieval, and the OASIS-MRI database^1^ [56] for MRI image retrieval. Figures 6, 7, 8, and 9 respectively show sample of images in each class. The four databases used in our experiments are summarized in Table 3 in terms of image number, size of each image, class number, and images in each class.

**Table 3:** Databases summary used in the experimental framework

S. No.	Database	No. of Images	Image Size	No. of Classes	Images/Class
1	TCIA-CT	604	$512 \times 512$	8	$\lbrace 75, 50, 58, 140, 70, 92, 78, 41 \rbrace$
2	EXACT09-CT	675	$512 \times 512$	19	$\lbrace 36, 23, 30, 30, 50, 42, 20, 45, 50, 24, 28, 24, 35, 40, 50, 35, 30, 28, 55 \rbrace$
3	NEMA-CT	315	$512 \times 512$	9	$\lbrace 36, 18, 36, 37, 41, 30, 23, 70, 24 \rbrace$
4	OASIS-MRI	421	$512 \times 512$	4	$\lbrace 124, 102, 89, 106 \rbrace$

## Experimental Results

This section includes several experiments that demonstrate the efficacy of the proposed method RbQE and compare its results to those of existing methods listed in Table 2. The RbQE method applied two different searching techniques: a rapid search for each database class using one query image selected from the database’s image collection, where every image in the database is considered a query. Then, the final search is done using the final NQE (FNQE). Note that all searches are automated without user participation or suggestion, which is considered a strong point. The performance of the proposed method is compared to that of modern methods, whether deep learning-based or not.

### Retrieval Performance on TCIA-CT Database

**Fig. 10:**
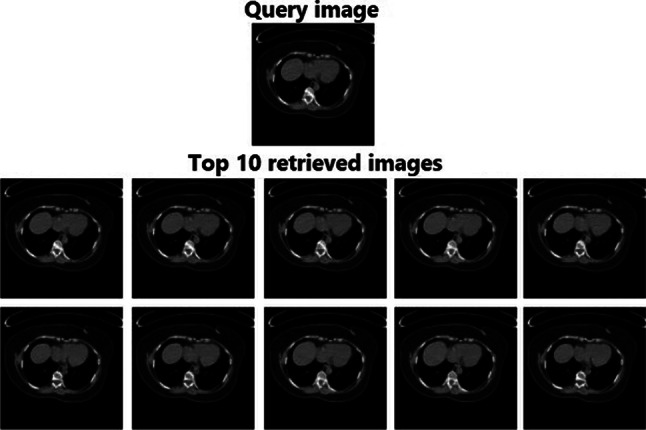
Retrieved images for a TCIA-CT database query using RbQE with VGG-19

The performance of the RbQE method on the TCIA-CT database was evaluated using two feature extractors, AlexNet and VGG-19, in addition to VGG-16, to demonstrate that VGG-19 with RbQE outperforms VGG-16 with RbQE. The retrieval results are shown in Table 4 in terms of ARP, ARR, and $F_score$. When compared to other methods, the suggested RbQE method using VGG-19 performs the best on the top 10 images. In terms of ARP, ARR, and $F_score$, the proposed method outperforms $ST-CCA_v$ by 0.84%, 0.16%, and 0.27%, respectively. Figure 10 exhibits the TCIA-CT database query outcomes of the RbQE method with VGG-19 features, which shows all the top 10 images in the same query image class.

**Table 4:** Performance of different methods on TCIA-CT database with the top 10 matches considered

Method	ARP	ARR	$F_{score}$
LBP	66.91	9.74	17.00
LMeP	73.71	10.77	18.79
LWP	88.40	13.09	22.80
SS3D	80.54	11.71	20.45
$HCSCs_{h}$	94.74	14.45	25.08
$HCSCs_{v}$	95.12	14.52	25.20
$ST-DC_{avg}$	95.80	14.65	25.41
$ST-DC_{max}$	95.71	14.58	25.30
$ST-CCA_{h}$	96.33	14.68	25.48
$ST-CCA_{v}$	96.45	14.71	25.52
$\mathbf {RbQE \ with \ VGG-16}$	$\mathbf {96.92}$	$\mathbf {14.80}$	$\mathbf {25.68}$
$\mathbf {RbQE \ with \ VGG-19}$	$\mathbf {97.29}$	$\mathbf {14.87}$	$\mathbf {25.79}$
$\mathbf {RbQE \ with \ AlexNet}$	$\mathbf {96.86}$	$\mathbf {14.77}$	$\mathbf {25.63}$

### Retrieval Performance on EXACT09-CT Database

**Fig. 11:**
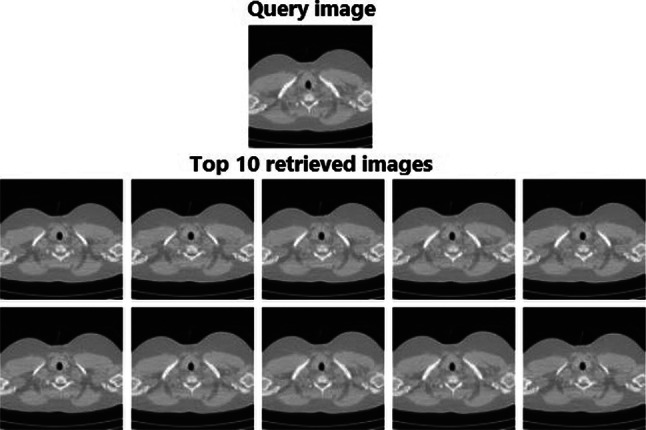
Retrieved images for an EXACT09-CT database query using RbQE with AlexNet

The comparison methods used in the “Retrieval Performance on TCIA-CT Database’’ section are also considered and evaluated here using the same experimental parameters as the TCIA-CT database. The retrieval result of the RbQE with different feature extractors is shown in Table 5. In comparison to other methods, the features of the AlexNet descriptor with the RbQE method achieve the highest performance on the top 10 images, and the result of the RbQE with VGG-16 exceeds the RbQE with VGG-19 only on that database, while all the descriptors with the RbQE method exceed the $ST-CCA_{v}$ method. The outcomes of the AlexNet with the RbQE method in relation to $ST-CCA_{v}$ in the ARP, ARR, and $F_{score}$ ranges are improved by 4.86%, 1.64%, 2.47%. The results of the top 10 images obtained using the RbQE search technique with AlexNet features are shown in Fig. 11.

**Table 5:** Performance of different methods on EXACT09-CT database with the top 10 matches considered

Method	ARP	ARR	$F_{score}$
LBP	65.03	19.51	30.02
LMeP	63.23	18.91	29.11
LWP	83.00	24.87	38.27
SS3D	67.00	20.09	30.91
$HCSCs_{h}$	90.74	28.53	43.41
$HCSCs_{v}$	91.50	28.83	43.84
$ST-DC_{avg}$	92.09	29.07	44.19
$ST-DC_{max}$	91.93	28.98	44.07
$ST-CCA_{h}$	91.92	28.90	43.98
$ST-CCA_{v}$	93.35	29.44	44.76
$\mathbf {RbQE \ with \ VGG-16}$	$\mathbf {98.16}$	$\mathbf {31.06}$	$\mathbf {47.19}$
$\mathbf {RbQE \ with \ VGG-19}$	$\mathbf {96.99}$	$\mathbf {30.63}$	$\mathbf {46.56}$
$\mathbf {RbQE \ with \ AlexNet}$	$\mathbf { 98.21}$	$\mathbf {31.08}$	$\mathbf {47.23}$

### Retrieval Performance on NEMA-CT Database

**Fig. 12:**
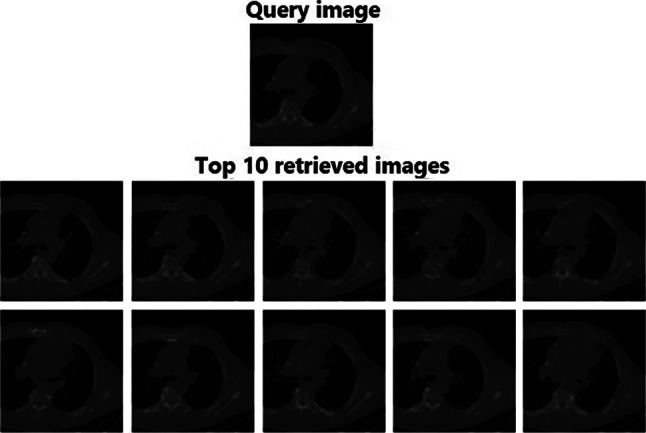
Retrieved images for a NEMA-CT database query using RbQE with VGG-19

We also use the NEMA-CT database to evaluate the performance of RbQE with different feature extractors and other modern methods. The proposed RbQE with VGG-19 features achieves the most satisfactory accuracy on the top 10 images and is superior to all other descriptors used by the RbQE. The retrieval results of the RbQE method with VGG-19 are improved by 1.24%, 0.18%, and 0.36% compared to the HCSCs method, as shown in Table 6. The result of the top 10 images for the query using the RbQE method with VGG-19 features is shown in Fig. 12.

**Table 6:** Performance of different methods on NEMA-CT database with top 10 matches considered

Method	ARP	ARR	$F_{score}$
LBP	90.55	29.33	44.31
LTCoP	92.15	30.31	45.62
LMeP	93.09	30.62	46.08
LWP	95.32	31.33	47.16
SS3D	92.24	30.26	45.57
LTP	92.00	30.23	45.51
LDP	94.22	31.08	46.74
LTrP	93.69	30.96	46.54
HCSCs	98.33	33.64	50.13
$\mathbf {RbQE \ with \ VGG-16}$	$\mathbf {99.14}$	$\mathbf {33.56}$	$\mathbf {50.15}$
$\mathbf {RbQE \ with \ VGG-19}$	$\mathbf {99.57}$	$\mathbf {33.82}$	$\mathbf {50.49}$
$\mathbf {RbQE \ with \ AlexNet}$	$\mathbf {99.44}$	$\mathbf {33.75}$	$\mathbf {50.39}$

### Retrieval Performance on OASIS-MRI Database

**Fig. 13:**
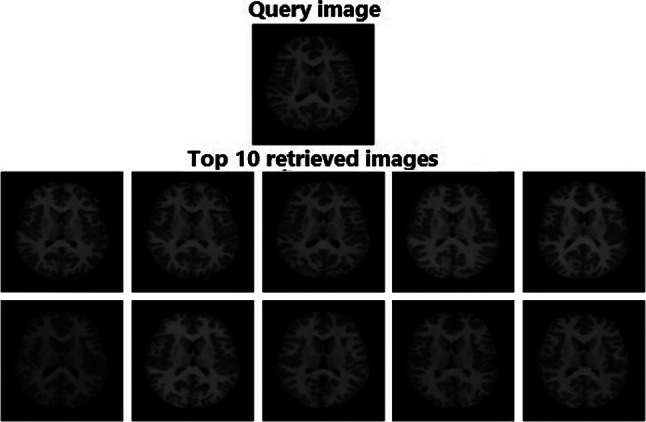
Retrieved images for an OASIS-MRI database query using RbQE with AlexNet

The efficiency of the RbQE method with different feature extractors was also compared against another medical image retrieval method, IR-Net [28], this method was tested using a benchmark database called Open Access Series (OASIS) with MRI [56]. We have followed all the settings for comparison as in IR-Net, where Table 8 presents the performance of the top 10 images in terms of ARP. In Table 7, the RbQE method with AlexNet, VGG-16, and VGG-19 features exceeds other existing methods as shown group-wise in terms of ARP. On the top 10 images, the RbQE with AlexNet features performs with the highest level of accuracy compared to the RbQE with VGG-16 and VGG-19. The retrieval results of the proposed method are improved by 14.51% on average group-wise compared with the IR-Net method. The results of the query using the RbQE method with AlexNet features are shown in Fig. 13.

**Table 7:** Performance of different methods on OASIS database in terms of ARP for group-wise

Method	Group1	Group2	Group3	Group4	Average
LBP	55.08	35.20	32.70	51.60	43.64
LTCoP	50.08	41.08	34.16	55.19	45.12
SS3D	44.19	39.02	35.73	41.42	40.08
LTP	52.90	37.06	35.73	51.32	44.25
LTrP	52.26	37.35	34.04	43.21	41.75
MDMEP	69.52	50.59	48.31	77.64	62.49
AlexNet	68.87	43.73	41.01	74.72	57.08
ResNet	69.11	45.59	44.27	66.42	56.35
VGG-16	70.73	44.61	38.09	61.04	53.62
IR-Net	77.10	55.29	57.19	88.40	69.49
$\mathbf {RbQE \ with \ VGG-16}$	$\mathbf {91.21}$	$\mathbf {79.41}$	$\mathbf {60.79}$	$\mathbf {83.11}$	$\mathbf {78.63}$
$\mathbf {RbQE \ with \ VGG-19}$	$\mathbf {91.45}$	$\mathbf {80.88}$	$\mathbf {62.25}$	$\mathbf {83.96}$	$\mathbf {79.64}$
$\mathbf {RbQE \ with \ AlexNet}$	$\mathbf {94.44}$	$\mathbf {76.67}$	$\mathbf {76.97}$	$\mathbf {87.92}$	$\mathbf {84.00}$

**Table 8:** Performance of different methods on OASIS database in terms of ARP for top 10 matches

Method	Top1	Top2	Top3	Top4	Top5	Top6	Top7	Top8	Top9	Top10
LBP	100	72.92	61.20	56.89	53.25	50.04	48.63	47.21	45.87	44.66
LTCoP	100	73.87	62.79	57.66	54.49	51.58	49.37	47.57	46.93	45.82
SS3D	100	68.53	56.77	51.54	47.89	45.68	43.77	42.37	41.44	40.45
LTP	100	73.63	63.42	57.84	53.87	51.46	49.37	47.77	46.45	45.04
LTrP	100	70.67	59.62	52.55	48.69	47.43	45.81	44.30	43.34	42.52
MDMEP	100	81.47	73.87	70.19	67.84	66.71	65.52	63.9	63.37	62.49
AlexNet	100	80.88	73.56	68.41	65.46	62.98	61.83	60.54	59.46	58.36
ResNet	100	78.50	71.18	67.99	64.94	62.87	60.54	59.26	58.38	57.48
VGG-16	100	76.60	68.88	63.84	61.24	59.62	58.23	57.60	56.03	55.06
IR-Net	100	83.37	78.62	76.37	74.68	73.44	72.21	71.62	70.89	70.45
$\mathbf {RbQE \ with \ VGG-16}$	$\mathbf {100}$	$\mathbf {100}$	$\mathbf {99.92}$	$\mathbf {98.22}$	$\mathbf {94.54}$	$\mathbf {91.05}$	$\mathbf {87.72}$	$\mathbf {84.68}$	$\mathbf {81.13}$	$\mathbf { 79.88}$
$\mathbf {RbQE \ with \ VGG-19}$	$\mathbf {100}$	$\mathbf {100}$	$\mathbf {99.92}$	$\mathbf {98.28}$	$\mathbf {95.01}$	$\mathbf {91.61}$	$\mathbf {88.77}$	$\mathbf {85.69}$	$\mathbf {83.27}$	$\mathbf {80.83}$
$\mathbf {RbQE \ with \ AlexNet}$	$\mathbf {100}$	$\mathbf {100}$	$\mathbf {100}$	$\mathbf {99.23}$	$\mathbf {97.1}$	$\mathbf {93.98}$	$\mathbf {91.58}$	$\mathbf {88.81}$	$\mathbf {86.8}$	$\mathbf {84.79 }$

### Time Complexity

The feature extraction average time, retrieval average time, and total CPU time in seconds are shown in Table 9 using the proposed RbQE with the different feature extraction methods (VGG-16, VGG-19, and AlexNet) over each database (TCIA-CT, EXACT09-CT, NEMA-CT, and OASIS-MRI). All experiments were carried out on a computer equipped with an Intel(R) Core(TM) i7-4510U processor running at 2.00 GHz, 8 GB of RAM, and a 64-bit Windows 10 Enterprise LTSC operating system. The total CPU time of AlexNet is less than VGG-16 and VGG-19. The retrieval times of VGG-16, VGG-19, and AlexNet are equal on the same database because they have the same dimension of feature vectors (4096).

**Table 9:** CPU elapse time (sec) for proposed RbQE with the different features extractions methods over all four test databases

Database	Feature Extraction Method	Feature Extraction Time (A) (sec)	Retrieval Time (B) (sec)	Total CPU time (A+B) (sec)
TCIA-CT	VGG-16	1.69	3.99	5.68
	VGG-19	1.84	3.99	5.83
	AlexNet	1.09	3.99	5.08
EXACT09-CT	VGG-16	1.69	4.93	6.62
	VGG-19	1.84	4.93	6.77
	AlexNet	1.09	4.93	6.02
NEMA-CT	VGG-16	1.69	2.62	4.31
	VGG-19	1.84	2.62	4.46
	AlexNet	1.09	2.62	3.71
OASIS-MRI	VGG-16	1.69	2.5	4.19
	VGG-19	1.84	2.5	4.34
	AlexNet	1.09	2.5	3.59

## Discussion

As we described earlier in the methodology’s main framework, there are two search processes: a rapid search using a single query image chosen at random from each class of images, followed by a final search utilizing newly expanded queries. One of the key advantages of our proposed method is that the images retrieved from this search are used as input for the expansion process automatically, without any user intervention or suggestion. The newly created query images will then be utilized in the final search, and all evaluation metrics will be produced based on the results of this search. Our proposed method has been proven to be superior in its retrieval ability in comparison to all the existing and state-of-the-art methods. Our method depends on improving three basic processes in the CBMIR framework to improve the medical image retrieval process: feature extraction, similarity measurement, and query expansion. Firstly, in the process of extracting features from medical images, we focus on extracting deep and high-level features able to represent the medical images with high accuracy, especially since medical images contain more details than natural images. These details are difficult to represent using the local descriptors that extract low-level features, leading to an increase in the problem of the semantic gap that occurs between both the visual input of the human visual system (HVS) and the system of imaging when lost information in the process of representation of the image is converted from high-level semantics to low-level features to reduce this problem and achieve high accuracy. We have focused on using deep learning descriptors that produce high-level features for medical images, so we found that pre-trained DCNN models can be used to achieve these goals. After testing and comparing many pre-trained DCNN models, we found that AlexNet and VGGNets achieve high accuracy in representing medical images. According to its characteristics, as we mentioned before, the AlexNet achieves high accuracy in representing the most complex and difficult datasets in representation (EXACT09-CT and OASIS-MR) because of the high similarity between classes and complex details inside the images, as shown in Figs. 7 and 9 respectively. For VGGNets, we found that VGG-19 provided a better representation of the datasets (TCIA-CT and NEAM-CT) than VGG-16 and AlexNet. Secondly, in the process of similarity measurement, the Euclidean distance (ED) has been used in other CBMIR methods, but we want to find if there is any other similarity measurement method that can enhance the result with us, so we have tested many methods such as Euclidean distance, Manhattan distance, and chi-square distance. Then, in the end, we found that ED achieves high accuracy in similarity measurement and enhancement of the result. Thirdly, the process of query expansion is considered the core of the RbQE method, where this process has two parts: the first part is to obtain the NQE for deep features of the top 10 images from each class for enhancement of the retrieval process, and the second part is to calculate the similarity between the original query and all NQEs for obtaining the most similarly formed NQE for the original query, which means that the original query is from the same class as that NQE, and then this NQE will be used in the final search, which leads to enhancement of the retrieval process. On the other hand, retrieval accuracy is comparatively more crucial for medical retrievals, particularly for diagnostic purposes, than implementation efficiency. Therefore, our method improved these two factors, high retrieval accuracy and low time consumption in implementation.

## Conclusion

In this paper, we proposed an efficient method (RbQE ) for the retrieval of medical images. Our method relies on expanding the query image with a fully automatic process by reformulating it based on calculating the mean value of the top-ranking images from each class. DCNNs (AlexNet and VGG-19) have been used as extractors of deep and high-level features. Our method has been tested on four publicly available databases with different formats (TCIA-CT, EXACT09-CT, NEMA-CT, and OASIS-MRI), and the results showed that our method achieved high accuracy compared to other state-of-the-art CBMIR methods.

## Footnotes

1 https://www.oasis-brains.org

## References

[1] Owais M, Arsalan M, Choi J, Park KR (2019). Effective Diagnosis and Treatment through Content-Based Medical Image Retrieval (CBMIR) by Using Artificial Intelligence. Journal of Clinical Medicine..

[2] Tschandl P, Argenziano G, Razmara M, Yap J (2018). Diagnostic accuracy of content–based dermatoscopic image retrieval with deep classification features. British Journal of Dermatology..

[3] Sadeghi M, Chilana P, Yap J, Tschandl P, Atkins MS. Using content-based image retrieval of dermoscopic images for interpretation and education: A pilot study. Skin Research and Technology. 2019 dec;26(4):503–512. 10.1111/srt.12822 10.1111/srt.12822 31845429

[4] Shinde A, Rahulkar A, Patil C. Content based medical image retrieval based on new efficient local neighborhood wavelet feature descriptor. Biomedical Engineering Letters. 2019 may;9(3):387–394. 10.1007/s13534-019-00112-0. 10.1007/s13534-019-00112-0 PMC6694329 31456898

[5] Kaur P, Singh RK. A Panoramic View of Content-based Medical Image Retrieval system. In: 2020 International Conference on Intelligent Engineering and Management (ICIEM). IEEE; 2020.

[6] Rui Y, Huang TS, Chang SF (1999). Image retrieval: Current techniques, promising directions, and open issues. Journal of visual communication and image representation..

[7] Smeulders AWM, Worring M, Santini S, Gupta A, Jain R (2000). Content-based image retrieval at the end of the early years. IEEE Transactions on Pattern Analysis and Machine Intelligence..

[8] Kokare M, Chatterji BN, Biswas PK (2002). A Survey on Current Content based Image Retrieval Methods. IETE Journal of Research..

[9] Liu Y, Zhang D, Lu G, Ma WY (2007). A survey of content-based image retrieval with high-level semantics. Pattern Recognition..

[10] Müller H, Michoux N, Bandon D, Geissbuhler A (2004). A review of content-based image retrieval systems in medical applications—clinical benefits and future directions. International Journal of Medical Informatics..

[11] Ahmad J, Sajjad M, Mehmood I, Rho S, Baik SW (2015). Saliency-weighted graphs for efficient visual content description and their applications in real-time image retrieval systems. Journal of Real-Time Image Processing..

[12] Ahmad J, Sajjad M, Rho S, Baik SW (2016). Multi-scale local structure patterns histogram for describing visual contents in social image retrieval systems. Multimedia Tools and Applications..

[13] Pölsterl S, Conjeti S, Navab N, Katouzian A (2016). Survival analysis for high-dimensional, heterogeneous medical data: Exploring feature extraction as an alternative to feature selection. Artificial intelligence in medicine..

[14] Brea MLS, Rodríguez NB, Maroño NS, González AM, García-Resúa C, Fernández MJG (2016). On the development of conjunctival hyperemia computer-assisted diagnosis tools: Influence of feature selection and class imbalance in automatic gradings. Artificial Intelligence in Medicine..

[15] Felipe JC, Traina AJM, Traina C. Retrieval by content of medical images using texture for tissue identification. 10.1109/cbms.2003.1212785.

[16] Unay D, Ekin A, Jasinschi RS (2010). Local Structure-Based Region-of-Interest Retrieval in Brain MR Images. IEEE Transactions on Information Technology in Biomedicine..

[17] Ojala T, Pietikäinen M, Harwood D (1996). A comparative study of texture measures with classification based on featured distributions. Pattern Recognition..

[18] Srensen L, Shaker SB, de Bruijne M (2010). Quantitative Analysis of Pulmonary Emphysema Using Local Binary Patterns. IEEE Transactions on Medical Imaging..

[19] Peng SH, Kim DH, Lee SL, Lim MK (2010). Texture feature extraction based on a uniformity estimation method for local brightness and structure in chest CT images. Computers in Biology and Medicine..

[20] Murala S, Wu QMJ (2013). Local ternary co-occurrence patterns: A new feature descriptor for MRI and CT image retrieval. Neurocomputing..

[21] Murala S, Wu QMJ (2014). Local Mesh Patterns Versus Local Binary Patterns: Biomedical Image Indexing and Retrieval. IEEE Journal of Biomedical and Health Informatics..

[22] Murala S, Wu QJ (2014). MRI and CT image indexing and retrieval using local mesh peak valley edge patterns. Signal processing: image communication..

[23] Rehman SU, Tu S, Huang Y, Yang Z. Face recognition: A novel un-supervised convolutional neural network method. In: 2016 IEEE International Conference of Online Analysis and Computing Science (ICOACS). IEEE; 2016.

[24] ur Rehman S, Tu S, Waqas M, Huang Y, ur Rehman O, Ahmad B, et al. Unsupervised pre-trained filter learning approach for efficient convolution neural network. Neurocomputing. 2019;365:171–190. 10.1016/j.neucom.2019.06.084.

[25] Dubey SR, Roy SK, Chakraborty S, Mukherjee S, Chaudhuri BB (2019). Local bit-plane decoded convolutional neural network features for biomedical image retrieval. Neural Computing and Applications..

[26] Lan R, Zhou Y (2017). Medical Image Retrieval via Histogram of Compressed Scattering Coefficients. IEEE Journal of Biomedical and Health Informatics..

[27] Lan R, Wang H, Zhong S, Liu Z, Luo X (2018). An integrated scattering feature with application to medical image retrieval. Computers & Electrical Engineering..

[28] Pinapatruni R, Bindu CS (2020). Learning image representation from image reconstruction for a content-based medical image retrieval. Signal, Image a nd Video Processing..

[29] Azad HK, Deepak A (2019). Query expansion techniques for information retrieval: A survey. Information Processing & Management..

[30] Houle ME, Ma X, Oria V, Sun J (2017). Query Expansion for Content-Based Similarity Search Using Local and Global Features. ACM Transactions on Multimedia Computing, Communications, and Applications..

[31] Kondylidis N, Tzelepi M, Tefas A (2018). Exploiting tf-idf in deep Convolutional Neural Networks for Content Based Image Retrieval. Multimedia Tools and Applications..

[32] Imbriaco R, Sebastian C, Bondarev E, de With P (2019). Aggregated Deep Local Features for Remote Sensing Image Retrieval. Remote Sensing..

[33] Chum O, Mikulik A, Perdoch M, Matas J. Total recall II: Query expansion revisited. 2011 jun. 10.1109/cvpr.2011.5995601.

[34] Gordo A (2020). Radenovic F.

[35] Feng B, Cao J, Chen Z, Zhang Y, Lin S. Multi-modal query expansion for web video search. 2010. 10.1145/1835449.1835583.

[36] Rashad M, Afifi I, Abdelfatah M. Content-based Medical Image Retrieval based on Deep Features Expansion. In: 2022 5th International Conference on Computing and Informatics (ICCI). IEEE; 2022. Available from: .

[37] Wainberg M, Merico D, Delong A, Frey BJ (2018). Deep learning in biomedicine. Nature Biotechnology..

[38] Deng J, Dong W, Socher R, Li LJ, Li K, Fei-Fei L. ImageNet: A large-scale hierarchical image database. 2009 June. 10.1109/cvpr.2009.5206848.

[39] Bar Y, Diamant I, Wolf L, Greenspan H. Deep learning with non-medical training used for chest pathology identification. 2015 Mar. 10.1117/12.2083124.

[40] van Ginneken B, Setio AAA, Jacobs C, Ciompi F. Off-the-shelf convolutional neural network features for pulmonary nodule detection in computed tomography scans. 2015 Apr. 10.1109/isbi.2015.7163869.

[41] Sermanet P, Eigen D, Zhang X, Mathieu M, Fergus R, LeCun Y. Overfeat: Integrated recognition, localization and detection using convolutional networks. arXiv preprint arXiv:1312.6229. 2013.

[42] Bar Y, Diamant I, Wolf L, Lieberman S, Konen E, Greenspan H (2016). Chest pathology identification using deep feature selection with non-medical training. Computer Methods in Biomechanics and Biomedical Engineering: Imaging & Visualization..

[43] Ciompi F, de Hoop B, van Riel SJ, Chung K, Scholten ET, Oudkerk M (2015). Automatic classification of pulmonary peri-fissural nodules in computed tomography using an ensemble of 2D views and a convolutional neural network out-of-the-box. Medical Image Analysis..

[44] Tan X, Triggs B. Enhanced Local Texture Feature Sets for Face Recognition Under Difficult Lighting Conditions. IEEE Transactions on Image Processing. 2010;19(6):1635–50. 10.1109/tip.2010.2042645. 10.1109/TIP.2010.2042645 20172829

[45] Zhang B, Gao Y, Zhao S, Liu J (2010). Local Derivative Pattern Versus Local Binary Pattern: Face Recognition With High-Order Local Pattern Descriptor. IEEE Transactions on Image Processing..

[46] Murala S, Maheshwari RP, Balasubramanian R (2012). Local Tetra Patterns: A New Feature Descriptor for Content-Based Image Retrieval. IEEE Transactions on Image Processing..

[47] Krizhevsky A, Sutskever I, Hinton GE (2012). Imagenet classification with deep convolutional neural networks. Advances in neural information processing systems..

[48] Simonyan K, Zisserman A. Very deep convolutional networks for large-scale image recognition. arXiv preprint http://arxiv.org/abs/1409.1556

[49] Dubey SR, Singh SK, Singh RK (2015). Local Wavelet Pattern: A New Feature Descriptor for Image Retrieval in Medical CT Databases. IEEE Transactions on Image Processing..

[50] Murala S, Wu QMJ (2015). Spherical symmetric 3D local ternary patterns for natural, texture and biomedical image indexing and retrieval. Neurocomputing..

[51] He K, Zhang X, Ren S, Sun J. Deep Residual Learning for Image Recognition. In: 2016 IEEE Conference on Computer Vision and Pattern Recognition (CVPR). IEEE; 2016.

[52] Galshetwar GM, Waghmare LM, Gonde AB, Murala S (2018). Multi-dimensional multi-directional mask maximum edge pattern for bio-medical image retrieval. International Journal of Multimedia Information Retrieval..

[53] Clark K, Vendt B, Smith K, Freymann J, Kirby J, Koppel P (2013). The Cancer Imaging Archive (TCIA): Maintaining and Operating a Public Information Repository. Journal of Digital Imaging..

[54] Lo P, van Ginneken B, Reinhardt JM, Yavarna T, de Jong PA, Irving B (2012). Extraction of Airways From CT EXACT-09). IEEE Transactions on Medical Imaging..

[55] NEMA-CT image database. [Online]; 2012. Available from: ftp://medical.nema.org/medical/Dicom/Multiframe/CT.

[56] Marcus DS, Fotenos AF, Csernansky JG, Morris JC, Buckner RL (2010). Open Access Series of Imaging Studies: Longitudinal MRI Data in Nondemented and Demented Older Adults. Journal of Cognitive Neuroscience..

